# Assessment of In Vitro Bioaccessibility of Polyphenols from Annurca, Limoncella, Red Delicious, and Golden Delicious Apples Using a Sequential Enzymatic Digestion Model

**DOI:** 10.3390/antiox10040541

**Published:** 2021-03-30

**Authors:** Giulia Graziani, Anna Gaspari, Claudio Di Vaio, Aurora Cirillo, Carolina Liana Ronca, Michela Grosso, Alberto Ritieni

**Affiliations:** 1 Department of Pharmacy, University of Naples Federico II, Via Domenico Montesano 49, 80131 Naples, Italy; annagaspari@virgilio.it (A.G.); carolinaliana.ronca@unina.it (C.L.R.); alberto.ritieni@unina.it (A.R.); 2 Department of Agricultural Sciences, University of Naples Federico II, Via Università 100, 80055 Portici, Italy; claudio.divaio@unina.it (C.D.V.); aurora.cirillo@unina.it (A.C.); 3 Department of Molecular Medicine and Medical Biotechnology, School of Medicine, University of Naples “Federico II”, CEINGE-Biotecnologie Avanzate, 80131 Naples, Italy; michela.grosso@unina.it; 4 Unesco Chair for Health Education and Sustainable Development, 80131 Naples, Italy

**Keywords:** apple, polyphenols, bioaccessibility, colon, antioxidant activity

## Abstract

Four different varieties of apples have been considered (Limoncella, Annurca, Red Delicious, and Golden Delicious) to estimate the extent of colon polyphenolics release after in vitro sequential enzyme digestion. Since several studies report a positive effect of apple polyphenols in colonic damage, we found of interest to investigate the colon release of polyphenols in different varieties of apples in order to assess their prevention of colonic damage. UHPLC-HRMS analysis and antioxidant activity (ABTS, DPPH, and FRAP assays) were carried out on the apple extracts (peel, flesh, and whole fruit) obtained from not digested samples and on bioaccessible fractions (duodenal and colon bioaccessible fractions) after in vitro digestion. Polyphenolic content and antioxidant activities were found to vary significantly among the tested cultivars with Limoncella showing the highest polyphenol content accompanied by an excellent antioxidant activity in both flesh and whole fruit. The overall trend of soluble antioxidant capacity from the soluble duodenal phase (SDP) and soluble colonic phase (SCP) followed the concentrations of flavanols, procyandinis, and hydroxycinnamic acids under the same digestive steps. Our results highlighted that on average 64.2% of the total soluble antioxidant activity was released in the SCP with Limoncella exhibiting the highest values (82.31, 70.05, and 65.5%, respectively for whole fruit, flesh, and peel). This result suggested that enzymatic treatment with pronase E and viscozyme L, to reproduce biochemical conditions occurring in the colon, is effective for breaking the dietary fiber-polyphenols interactions and for the release of polyphenols which can exercise their beneficial effects in the colon. The beneficial effects related to the Limoncella consumption could thus be of potential great relevance to counteract the adverse effects of pro-oxidant and inflammatory processes on intestinal cells.

## 1. Introduction

Apple is among the most produced and consumed fruits worldwide. Numerous studies in the literature report the health effects of phytonutrients contained in this fruit [1,2]. The health benefits of apples are widely attributed to polyphenolic compounds that represent a group of secondary metabolites with aromatic rings bearing one or more hydroxyl groups. There are five main groups of polyphenolic compounds found in apple fruits: phenolic acids (chlorogenic acid and its derivatives), flavanols (catechin, epicatechin and procyanidins), flavonols (quercetin glycosides), dihydrochalcone (phloretin glycosides), and anthocyanins (cyanidin and its gycosides). These compounds act as effective antioxidants by protecting cell walls from free radical damage and by inhibiting the oxidation of low-density lipoproteins [3,4,5]. In general, human and animal study results showed that apple consumption improved lipid metabolism, metabolic dysfunctions such as hyperglycemia and insulin resistance [6,7,8]. In addition, many studies report the effects of apples on blood lipid profile, gastrointestinal health, and antioxidant status [9,10,11]. On the other hand, it is well-known that the quali-quantitative profile of polyphenolic compounds as well as the antioxidant potential in apples depend on a number of factors such as harvest time, cultivar, cultivation area, and storage conditions [12,13,14,15].

Gastro-intestinal digestion affects polyphenols and their stability and this, in turn, will affect their bioaccessibility and their potential effects on the intestinal cells. Koutsos et al. [16] have demonstrated that a significant percentage of apple polyphenols are not absorbed in the small intestine and together with the non-digestible polysaccharides reach the colon, where they can interact with the gut microbiota.

In an in vitro study, apple proanthocyanidins have been shown to be converted into phenylpropionic, phenylacetic, and benzoic acid derivatives by the colon bacteria [17]. In contact with the gut microbiota, polyphenols undergo a biotransformation that enhance their bioavailability. Moreover, polyphenols and their metabolic products modulate the gut microbiota composition by inhibiting the pathogenic bacteria and stimulating beneficial bacteria, therefore acting as potential prebiotics [16]. The interactions between dietary polyphenolic compounds and intestinal microbiota are therefore crucial for the health of the human host. There are few in vitro studies investigating the effects of whole apples on the intestinal comfort, simulating the colon digestion process and only a recent study simulates this digestive step using human fecal inoculum.

The aim of the current work was to assess the colon bioaccessibility of whole apples, peel and flesh belonging to four different cultivars (Annurca, Limongella, Red Delicious, and Golden Delicious) by simulating as much as possible the conditions of this intestinal compartment.

In particular, as we had reported in a previous study [18], colon digestion stage was simulated using a mix of bacterial enzymes, such as Pronase E and Viscozyme L (mix of bacterial protease and carbohydrases, respectively). The combination of Pronase E and Viscozyme L reproduces the biochemical conditions physiologically occurring in the colon, simulating the action of microbiota on the digested dietary matrix. Additionally, the polyphenolic quali-quantitative profile by UHPLC-Q-Orbitrap HRMS, total polyphenolic content, and antioxidant capacity of apple fruits (before simulated in vitro digestion) were investigated.

## 2. Materials and Methods

### 2.1. Plant Material

The trial was carried out in 2020 on apple fruits of four cultivars (Limoncella, Annurca traditional, Golden Delicious and Red Delicious) from a private orchard located about 200 m above sea level of the province of Avellino (Italy). For each cultivar, fruit samples were harvested from six trees in full production, trained to free palmette, grafted on M26, and planted at 3.0 m within rows and 5.0 m between rows. For Gold Deliciuos and Red Delicious cultivars, fruits were harvested in the second decade of September, while for the other two cultivars (Annurca traditional and Limoncella) during the first decade of October. The apple fruits of the Annurca traditional cultivar were then laid on the ground in specially constructed “melai” for a 15 days period to complete the redness. Then, the apple fruits of all cultivars were cold stored at 2 °C up to 2 months and analyzed.

### 2.2. Chemical Analysis of Fruits

Ten fruits per cultivar were sampled and analyzed for soluble solids content (SSC), pH, and titratable acidity (TA). The SSC (°Brix) of the juice extracted from each fruit was determined using a digital refractometer (HI 96814, Hanna Instruments, Villafranca Padovana, Italy), equipped with a temperature compensation system and the data were expressed as °Brix. TA (g malic acid per 100 g FW) was determined by neutralizing fruit juice acids with an alkaline solution (0.1 mol L^−1^ NaOH) to a final pH value of 8.2 (Orion 2-Star Benchtop pH Meter, Thermo Electron Corporation, Waltham, MA, USA).

### 2.3. Reagents and Materials

The standard of polyphenols (purity ≥ 98%), including chlorogenic acid, caffeic acid, rutin, catechin, epicatechin, phloridzin, kaempferol 3-O-glucoside, apigenin glucoside, phloretin, procyanidin b1, and procyanidin b2, were purchased from Sigma–Aldrich Chemical Co. (St. Louis, MO, USA). Reagents used for the antioxidant tests such as gallic acid, 6-hydroxy-2,5,7,8-tetramethylchromane-2-carboxylic acid (Trolox), 1,1-diphenyl-2-picrylhydrazyl (DPPH), 2,3,5-triphenyltetrazolium chloride (TPTZ), anhydrous ferric chloride, hydrochloric acid, and sodium acetate were purchased from Sigma–Aldrich (Milan, Italy). Methanol (MeOH) and water (LC-MS grade) were purchased from Carlo Erba reagents (Milan, Italy), whereas formic acid (98–100%) was purchased from Fluka (Milan, Italy).

### 2.4. Polyphenols Extraction

Peel and pulp were obtained from ten randomly selected apples for each cultivar. Each fruit was divided into four parts and carefully peeled with a stainless steel vegetable peeler to obtain the peel without flesh. The remaining flesh was cut into small pieces discarding the core and the seed. The whole fruit was obtained from five randomly selected apples for each cultivar. Each apple was divided into four parts, the core and seeds were eliminated and finally the resulting sample was cut into small pieces. Peel, flesh, and whole fruit, then, were frozen and subsequently freeze-dried. Polyphenols were extracted by ultrasound-assisted extraction on lyophilized samples according to a previous reported method [19]. Before extraction, lyophilized samples were ground in a mill IKA A11 (IKAWerke, Staufen, Germany) and 0.5 g of each sample was used for polyphenols extraction. The extracts obtained were then filtered through 0.22 µm nylon filters (Phenomenex, Castel Maggiore, Italy), prior to injection into the UHPLC-Orbitrap MS. The same extracts were used for antioxidant capacity and total polyphenolic content determinations.

### 2.5. UHPLC-Q-Orbitrap HRMS Analysis

A UHPLC system (UHPLC, Thermo Fisher Scientific, Waltham, MA, USA) was used for quantification and separation of polyphenolic compounds. A Q Exactive Orbitrap LC–MS/MS (Thermo Fisher Scientific, Waltham, MA, USA) was employed to facilitate the analysis of the mass spectrometry. The details of UHPLC-high-resolution mass spectrometry analysis are as described by Graziani et al. [20].

### 2.6. Determination of Antioxidant Activity

The free radical scavenging activity of the polyphenolic extracts was analyzed using 2,2-diphenyl-1-picryl-hydrazyl (DPPH) according to Brand-Williams et al. [21] with few modifications. The DPPH solution was prepared in methanol (4 mg in 10 mL) and diluted with the same solvent to obtain an absorbance value of 0.90 (±0.02) at 517 nm (DPPH radical working solution). The radical scavenging activity of the apple extracts was determined by adding l mL of DPPH radical working solution and 200 µL of suitably diluted apple extract. The decrease in absorbance of the resulting solution was monitored at 517 nm after 10 min of incubation and the results were expressed in TEAC (mmol Trolox equivalents per kg dry weight of sample). All determinations were performed in triplicate.

The ferric reducing antioxidant power was determined using a FRAP assay [22] with minor modifications. Briefly, the FRAP reagent contained 1.25 mL of 10 mM TPTZ (2,4,6-tripyridyl-striazine) in HCl (40 mM), 1.25 mL of FeCl_3_ (20 mmol) in water, and 12.5 mL of sodium acetate buffer (0.3 M, pH 3.6). The apple extracts (150 µL) were allowed to react with 2.850 mL of FRAP reagent. The absorbance was monitored after 4 min at 593 nm. The results were expressed as TEAC (mmol Trolox equivalents per kg dry weight of sample). All the determinations were performed in triplicate.

The ABTS-scavenging capacity assay was carried out according to the methodology proposed by [23] with minor modifications. A stock was prepared with 2.5 mL of aqueous ABTS (7 mM) and 44 mL of 2.45 mM of potassium persulfate was rested from 12 h to 16 h at room temperature. The ABTS solution was diluted with absolute ethanol until reaching an absorbance of (0.700 ± 0.002) nm to obtain an ABTS radical working solution. After that, 100 mL of sample and 1 mL of the above resulting solution were added. After 3 min, the absorbance was measured at 734 nm. The results were expressed as TEAC (mmol Trolox equivalents per kg dry weight of sample). All determinations were performed in triplicate.

### 2.7. Total Polyphenolic Content Determination

The content of total phenolics was determined according to a Folin–Ciocalteu method [24], with slight modifications. Briefly, 125 µL of appropriately diluted extract was mixed with 500 µL of deionized water and 125 µL of the Folin–Ciocalteu reagent for 6 min at room temperature. Subsequently, 1 mL of deionized water and 1.25 mL of 7.5% of sodium carbonate solution were added to the mixture. After 90 min of incubation in the dark, the absorbance at 760 nm was measured. Concentrations of total phenolic were expressed in terms of mg of gallic acid equivalents (GAE) per gram of dry weight (DW), based on a standard linear curve (R^2^ > 0.995) that was computed over a dynamic range 0.05–2.5 g/L gallic acid. Each extract was analyzed in triplicate.

### 2.8. In Vitro Sequential Enzyme Digestion

Total of 2.5 g of freeze-dried apple (flesh, peels, and whole fruits) and 2.5 g of cellulose (control) were subjected, separately, to simulated in vitro oral, gastric, pancreatic, and colonic digestion following a previously described method [25] with few modifications. In particular, apple tissues were suspended in 1.75 mL of simulated salivary fluid. After 1 min of stirring, 0.25 mL of α-amylase solution (made up in simulated salivary fluid, 75 U/mL) was added followed by 12.5 µL of 0.3 M calcium chloride, and 488 µL of water.

Then, the pH of the mixture was adjusted to 7 with HCl 1 M and the solution was incubated in a shaker bath (100 cycles/min) at 37 °C for 2 min. The gastric phase was simulated by adding to the oral bolus, 3.75 mL of simulated gastric fluid, 0.8 mL of pepsin solution (made up in simulated gastric fluid, 2000 U/mL), 2.5 µL of 0.3 M calcium chloride.

The pH of the solution was adjusted to 3 with HCl 1 M, the volume was filled up to 10 mL with distilled water and the mixture was incubated in a shaker bath (100 cycles/min) at 37 °C for 2 h. Then, in order to simulate the duodenal conditions, gastric digested was added with 5.5 mL of simulated intestinal fluid, 2.5 mL pancreatin solution (made up in simulated intestinal fluid, 100 U/mL of trypsin activity), 20 µL of 0.3 M calcium chloride, 1.25 mL bile salt solution (65 mg/mL), the mixture was thoroughly mixed, and the pH of the solution was adjusted to 7 with NaOH 1 M. Then, the volume was adjusted to 20 mL with water, the mixture was incubated in a shaker bath (100 cycles/min) at 37 °C for 2 h and then centrifuged at 4900× *g* at 37 °C for 10 min.

The supernatant fraction (after duodenal phase) was collected for the analysis, and the remaining insoluble material (pellet) was added with 15 mL of distilled water and 2.5 mL of 1 mg/mL Pronase E solution (pH 8), vortexed for 1 min and incubated at 37 °C for 1 h to simulate the post duodenal phase. After centrifugation (4900× *g* at 37 °C for 10 min), the supernatant was collected for the following analysis and the pellet was treated with 75 μL of Viscozyme L (pH 4), 17.5 mL of distilled water and vigorously vortexed for 1 min. Subsequently, samples were incubated newly at 37 °C for 16 h after that centrifuged at 4900× *g* at 24 °C for 10 min and the supernatant was collected for the analysis. The treatment with pronase was carried out to simulate the action of bacterial protease and is particularly suitable to hydrolyze insoluble material; Viscozyme L (cellulolytic enzyme mixture) was used, instead, to obtain the complete disruption of the apple flesh, peels, and whole fruit matrix [18].

### 2.9. Statistical Analysis

Statistical analyses were performed using the software Statistical Product and Service Solutions (SPSS 12.0.1). Statistical differences were evaluated through one-way analysis of variance (ANOVA). Tukey’s post hoc test was used for mean separation and the statistical significance of the comparisons was defined as *p* < 0.05.

## 3. Results and Discussion

### 3.1. Total Soluble Solids (TSS) Content, Total Acidity (TA), and pH of Fruits

The chemical analysis results for the apple cultivars investigated are presented in Table 1. The TSS is a good indicator of sugar content of apples and presumably of sweetness [26]. The Limoncella cultivar showed higher TSS values (16.07 °Brix) than cultivars Golden Delicious (11.93 °Brix), Annurca (11.73 °Brix) and Red Delicious (10.57°Brix). Titratable acidity (TA), instead, may be an important tool in predicting the taste of apples. This is important during the assessment of fruit quality, since consumers often have distinct preferences for acid or sweet tasting apples [27].

Also for the titratable acidity, the Limoncella cultivar reported statistically superior values equal to 7.57 g/L, while the Annurca and Golden Delicious cultivars did report value equal to 5.87 and 5.47 g/L respectively; lower acidity was shown in the cultivar Red Delicious with value equal to 3.03 g/L. Similar results have also been observed by Minnocci et al. [28] who showed that Limoncella cultivar had very high soluble solids (28.2 °Brix) and the highest acidity. This high sugar content is an interesting feature for an apple cultivar, both for its taste and for its effect on storage-life, as sugars are a cryoprotectant. Our results are consistent with those previously reported by Wu et al. [29] who showed that the summer variety, Delicious, showed relatively low levels of soluble solids, compared to the cultivars harvested in September and October, the level of soluble solids differed considerably. We also analyzed the pH variations in the apple juices, which showed significant differences between Red Delicious (4.07) and Golden Delicious (3.63) and Annurca (3.60); the Limoncella cv with 3.83 pH did not show significant statistical differences with Golden Delicious and Annurca cultivars.

### 3.2. Antioxidant Activity and Total Polyphenolic Content Measurements

The levels of phenolic compounds and their antioxidant activity are relevant aspects in the analysis of health promoting activity of apple cultivars. The radical scavenging activity was thus determined on apple extracts (peel, flesh, and whole fruit) obtained from not digested samples and on bioaccessible fractions (duodenal and colon bioaccessible fractions) obtained after sequential enzyme in vitro digestion. As the measurement of total antioxidant activity evaluated with different assays is important to get the overall antioxidant potential of any food matrix, in this study we used three well-known spectrophotometric assays to determine ABTS^+^ radical scavenging activity, DPPH free radical-scavenging activity, and ferric reducing antioxidant capacity (FRAP). The results are reported in Table 2 and are expressed as mmol trolox/kg dw.

With regard to the peel, in all the cultivars analyzed there was a higher content of total polyphenols and a higher antioxidant activity compared to the flesh and whole fruit. In agreement with our data, different researches reported that depending on cultivar, apple peel contains about two to nine times more total polyphenolic content than their pulp [30,31].

As for the flesh, the cultivar Limoncella was the one with the highest level (2.17 mg/g dw). The cultivar Annurca showed a content of 1.88 mg/g dw while there were no significant differences between the cultivars Red Delicious and Golden Delicious. These results are relatively comparable with those of other papers on apples, although it is unavoidable to have a broad variety of values for apples especially in reference to the cultivar, geographical area of cultivation, and environmental conditions [12,32].

Overall, the antioxidant capacity varied significantly (*p* < 0.05) among cultivars and was higher in the peel than in the flesh and whole fruit. These results well correlated with the total polyphenolic contents measured by the Folin method. Discrepancies between polyphenolic content and antioxidant activity were noted for the FRAP method, whereas ABTS and DPPH proved to be the most reliable methods [33,34]. On the other hand, not completely overlapping results were likely caused by synergistic effects between polyphenols and other chemical constituents such as ascorbic acid and beta-carotene that can contribute to the overall antioxidant activity. Moreover, it is also reported that some polyphenolic compounds show different antioxidant activity depending on the measurement method used [35].

In the whole apple fruit, the greatest total phenolics content and antioxidant activity was found in Limoncella cultivar, followed by Annurca and Red Delicious, whereas the lowest values were found in Golden Delicious. Higher antioxidant potential and content of total polyphenols in peel and in the whole fruit compared to the flesh fraction was described previously for other apple cultivars [13]. As shown in Table 2, whole fruit of Limoncella showed an antioxidant activity and a total phenolic content higher than Annurca which represents one of the traditional varieties from Southern Italy largely appreciated for its special taste and health properties [36].

These results are in accordance with the previous reports [36] which have shown that several traditional cultivars, including Limoncella, have a higher polyphenols content and antioxidant activity compared to more common cultivars such as Annurca. As we had previously reported [20], the total polyphenolic content measured with the Folin assay was slightly higher and not well correlated with the amount of total phenolics provided by UHPLC-HRMS data, for these reasons we used Folin results and UHPLC-HRMS data to evaluate, respectively, quantitative and qualitative cultivar effects.

### 3.3. Quali-Quantitative Polyphenolic Profile by UHPLC-Q-Orbitrap HRMS

Our results showed that procyanindins, flavanols, and flavonols are present in higher concentrations in peels ranging from 62 to 79% of the total polyphenols, while hydroxycinnamics and dihydrochalcones are present at lower concentration (58–6%). Among procyanidins reported in peels, the most representative were dimers (70–84%), trimers (14–26%), and tetramers (2–4%). The cultivar Limoncella is prominent for the high content of flavanols with levels of 903.13, 860.29, and 915.92 μg/g dw respectively in the flesh, peel, and whole fruit. Panzella et al. [37] carried out a study on traditional apple cultivars native of Campania Region and reported a higher content of flavanols and hydroxycinnamates for Limoncella than for Annurca [37]. Table 3 reports the phenolic composition obtained by UHPLC-HRMS analysis in flesh and peel of the four different apple cultivars and Table 4 shows the quali-quantitative polyphenolic profile of the flesh and whole fruits of the same cultivars.

Single phenolic compounds were quantified using calibration curves built with appropriate reference compounds. Coumaroyl quinic acid and epicatechin trimer and tetramer were quantified using calibration curves of chlorogenic acid and procyanidin b2, respectively. The quantification of polyphenolic compounds was also carried out by HRMS considering that the Folin assay may be affected by interferences such as ascorbic acid and reducing sugars. The investigated compounds were classified into five groups, such as hydroxycinnamic acids (chlorogenic acid, coumaroyl quinic acid, and caffeic acid), flavanols (catechin and epicatechin), flavonols (rutin, hyperoside, kaempferol-3-O-glucoside, apigenin-7-O-glucoside, and isorhamnetin derivatives), procyanidins (procyanidin B1, B2, trimer, and tetramer), and dihydrochalcones (phloretin, phloretin-2-O-xyloglucoside, and phloridzin). As reported by several authors, peel was richer than flesh in total polyphenols as well as the majority of the single polyphenolic compounds, in accordance with its defensive rule against pathogenic pressure which mainly acts on the skin [14,38]. The great variability in polyphenol content and profile observed among apple cultivars was in agreement with other studies [30,37,39]. The complex of flavonols was identified as quercetin, kaempferol, apigenin, and isorhamnetin derivatives in all the part of apple with significant predominance in peels (Table 4; Table 5). Among the quercetin derivatives, the most representative in peels was the hyperoside (quercetin-3-O-galactoside), for all analyzed cultivars, with Annurca that showed the highest content (468 mg/g dw) followed by Red Delicious (427.82 mg/g dw) and Golden Delicious (401.35 mg/g dw), while Limoncella showed the lowest value (213.77 μg/g dw). Dihydrochalcones also represent a considerable amount of apple polyphenols, especially in the peels, (10–13% of the polyphenolic content) according to previous published results [13,40].

The results reported for the flesh and the whole fruits (Table 4), in accordance with literature data [12,13,15], highlighted that the most representative polyphenols are hydroxycinnamic acids, flavanols, and procyanidins ranging from 85.81 to 93.80% of the total polyphenols, while flavonols represented a very low incidence as well as dihydrochalcones. The most representative compound in the flesh and in the whole fruit was chlorogenic acid which contributes 32–56% of the total polyphenols and even more interestingly with the cultivars Limoncella and Annurca showing the highest values (882.76 and 861.79 μg/g dw for flesh and 841.57 and 820.28 μg/g dw for whole fruits, respectively). As reported for peels, in flesh and whole fruits, catechin, epicatechin, and procyanidins (B1 and B2) were also present at high concentrations, and together accounted for ~32–59% of the whole; lower contributions were observed for flavonols whereas dihydrochalcones it is the less represented class of polyphenols reaching levels between 5–8%.

On the basis of the results obtained, it was therefore possible to define two main groups of apple cultivars. The first group comprises the cultivars with the higher amounts of flavanols and hydroxycinnamic acids in the peel and flesh (Limoncella and Annurca cultivars). The second group comprises the Red Delicious and Golden Delicious cultivars with the greater amounts of flavonols in the fruits (especially peels).

### 3.4. Bioaccessibility of Apple Polyphenols and Antioxidant Activity upon Digestion

Apples are considered an important source of antioxidant compounds, however, the intake of large quantities of these compounds present in a food matrix does not always produce a significative increase of their concentration in blood and tissues which depends mainly on the digestion and assimilation mechanisms of phytonutrients. Since several studies in the literature have reported the positive effects of apple polyphenols on the colon, in this study, we evaluated the colonic release of totally bioaccessible polyphenols by simulating in vitro digestion through a multistep enzymatic protocol. In particular, in vitro digestion was carried out using the INFOGEST protocol [24] that simulates gastric and duodenal conditions. Colon bioaccessibility was evaluated using pronase E to simulate the activity of bacterial proteases and a multi-component carbohydrase (Viscozyme L) to hydrolyze plant cell wall polysaccharide [18].

Table 6 summarizes the results of polyphenols bioaccessibility. Data showed that hydroxycinnamic acids were the most abundant compounds released from flesh and whole fruits in the SDP (soluble duodenal phase) and SCP (soluble colonic phase), the abundance being ~73% of total bioaccessible polyphenols whereas flavonols were the most widely released compounds in both the SDP and the SCP in the case of peels with an abundance of ~68% of total polyphenols. The results obtained show that ~17% of bioaccessible polyphenols from apple (whole and flesh) were flavonols while Annurca and Limoncella flesh showed a release of these compounds equal to ~1% in the SDP and SCP. On the other hand, in vitro digestion of Annurca and Limoncella flesh showed that ~78% of totally bioaccessible polyphenols were hydroxycinnamic acids while dihydrochalcones represented about 17% of totally bioaccessible polyphenols. About 54% of totally bioaccessible polyphenols were released in the SCP, and interestingly, flavanols and procyanidins were entirely released in in this intestinal compartment. Analysis of the colon bioaccessibility under in vitro gastrointestinal digestion (Table 7) revealed the highest release of polyphenolic compounds for the Limoncella cultivar with a release of 46.10, 67.59, and 71.96% respectively for whole fruit, flesh, and peel. Despite a part of polyphenols ranging between 28.04 and 66.32% were released in the duodenal digestion, a considerable amount was released at the colon level (an average 54% of totally bioaccessible polyphenols). This release is due to the action of enzyme such as Pronase E and Viscozyme L (including polysaccaridases such as arabanase, cellulase, β-glucanase, hemicellulase, and xylanase) on the unsolubilized pellet derived from the previous digestive step. Interestingly, the compounds mainly released in SCP were flavanols and procyanidins accounting for 4.22 and 2.93% of total polyphenols bioaccessible in the SCP.

A previous study had reported no detection of either procyanidin B2 or epicatechin after intestinal digestion leading to hypothesize the degradation to unknown products following the transition from the gastric to the intestinal environment [41]. It has been calculated that the procyanidin level retrieved only upon colon digestion, is ~0.87% of that present in the undigested apples demonstrating that a large amount of these compounds are unstable under digestion conditions and that the fraction delivered is bound to the apple insoluble fiber. Apples, in fact are a source of both soluble and insoluble fiber. Goristein at al. reported that the content of insoluble dietary fiber in apples is about 50% of the total [42]. This finding supports the hypothesis that non-extractable procyanidins associated with dietary fibers arrive intact to the colon and undergo the action of cell-wall degrading enzymes on the pellets resulting from the SDP [43].

Hydroxycinnamic acids were released in the range of 34.86 to 81.84% of totally bioaccessible polyphenols, with Limoncella exhibiting the highest values for whole fruit, flesh, and peel in comparison with other cultivars (Table 6). In particular, high resolution mass spectrometric analysis carried out on soluble fraction upon duodenal and colonic digestion highlighted a high release of chlorogenic and caffeic acid especially following the viscozyme treatment (Table 6). Caffeic and chlorogenic acid release could be in part explained by the breakdown of the linkage between these compounds and insoluble dietary fiber following the hydrolitic action of enzyme on cell wall as previously reported for artichoke and oat bran [44,45]. On the other hand, chlorogenic acid in vivo can be hydrolyzed by the gut microflora into various aromatic acid metabolites including caffeic acid and quinic acid [46]. From the health point of view, the potential release of chlorogenic and caffeic acid in the colon is noteworthy because these compounds exert a significant antioxidant activity leading to a decrease of oxidative cell damage in human colon cell lines [47]. With regard to flavonols and dihydrochalcones it is interesting to underline the highest release (88.30%) in the colon simulated digestion from Limoncella peels, while for other cultivars the release of these compounds in the colon was between 19.91 and 81.08%. The importance of the potential delivery of dihydrochalcones in the colon is widely supported by literature studies that report antioxidative, anti-inflammatory, antiproliferative, and pro-apoptotic properties of these compounds in human colon cancer Caco-2 and HT-29 cell lines [48,49]. The release of individual polyphenols after in vitro digestion is reported in the Supplementary Table S1.

The antioxidant activity of soluble fractions (SDP and SCP) evaluated by the DPPH method after duodenal and colonic digestion is reported in Table 8. The potential antioxidant activity of polyphenolic compounds from apples along the gastro-intestinal tract is in accordance with previous in vitro and in vivo evidences [9]. Noteworthy, a first set of experiments to evaluate these activities had also been conducted with the ABTS and FRAP assays although differently from the DPPH assay, these methods resulted to be affected by pH and enzymes interference.

The overall trend of soluble antioxidant capacity from the SDP and SCP followed the concentrations of flavanols, procyandinis and hydroxycinnamic acids under the same digestive steps. Interestingly, on average, 64.2% of the total soluble antioxidant activity was released in the SCP, with Limoncella exhibiting the highest values (82.31, 70.05, and 65.5%, respectively for whole fruit, flesh, and peel). This result suggested that enzymatic treatment with pronase E and viscozyme L responsible for the apple cell-wall breakdown released the so-called non-extractable polyphenols (catechins, procyanidins, chlorogenic, and caffeic acid) mainly bound to the polysaccharides of the dietary fiber matrix with an appreciable antioxidant capacity. Therefore, the antioxidant activity of soluble fractions (SDP and SCP) may play a role in the intestinal tract by maintaining the redox equilibrium against harmful oxidizing agents and preventing intestinal diseases related to the generation of oxygen free radicals during the digestion process.

## 4. Conclusions

Apples are a rich source of polyphenols and several studies have highlighted the positive effects of apple antioxidants on gut homeostasis. However different cultivars display variations in their phenolic profile and antioxidant activities. In this study, with the aim to explore the nutraceutical potential of different cultivars, we evaluated the antioxidant profiles of two widely grown apple varieties (Golden Delicious and Red Delicious) and two local cultivars from Campania region in Southern Italy (Limoncella and Annurca) by a mass spectrometry-based approach and in vitro simulation of gastrointestinal digestion. Extracts from different fruit components (peel, flesh, and whole fruit) were examined. Interestingly, polyphenolic content and antioxidant activities were found to vary significantly among these cultivars with Limoncella showing the highest polyphenol content accompanied by an excellent antioxidant activity in both flesh and whole fruit. Even more interestingly, the in vitro digestion processes showed higher values of bioaccessible polyphenols for Limoncella thus indicating that colon digestion is very effective in breaking dietary fiber-polyphenols interactions and releasing protective antioxidant activities for this cultivar. Therefore, although these results are based on an in vitro approach that does not take into account the complexity of the in vivo digestion process including the gut microbiota contribution to biotransformation and release of bioactive metabolites, our study provides experimental evidence regarding the effects of food matrix on polyphenol bioaccessibility and highlights the beneficial effects of Limoncella consumption that could be of potential great relevance to counteract the adverse effects of pro-oxidant and inflammatory processes on intestinal cells.

## Figures and Tables

**Table 1:** Fruit characteristics: total soluble solids (SSC), titratable acidity (TA) and pH. Means followed by different letters are significantly different for *p* ≤ 0.05. *, *** significant at *p* ≤ 0.05 and 0.001, respectively.

Cultivar	TSS (°Brix)	TA (g L^−1^ Malic Acid)	pH
Limoncella	16.07 ± 0.03 a	7.57 ± 0.32 a	3.83 ± 0.14 ab
Red Delicious	10.57 ± 0.33 c	3.03 ± 0.03 c	4.07 ± 0.05 a
Golden Delicious	11.93 ± 0.03 b	5.47 ± 0.61 b	3.63 ± 0.07 b
Annurca	11.73 ± 0.31 b	5.87 ± 0.30 b	3.60 ± 0.06 b
Significance	***	*	***

**Table 2:** Antioxidant activity (ABTS, DPPH, and FRAP) and total polyphenolic content (FOLIN) in Limoncella, Red Delicious, Golden Delicious, and Annurca apples. Each value represents the mean ± SD of three biological and two technical replicates. The same letter indicates not significant differences according to Tukey’s multiple range test (*** *p* < 0.05).

Cultivar	FOLIN	ABTS	DPPH	FRAP
	(mg/g dw)	(mmol Trolox/Kg dw)	(mmol Trolox/kg dw)	(mmol Trolox/kg)
		***Peel***		
Limoncella	2.29 ± 0.05 b	88.89 ± 3.30 c	28.21 ± 0.21 b	61.14 ± 1.76 a
Red Delicious	3.54 ± 0.07 a	131.95 ± 6.73 a	42.08 ± 1.05 a	48.08 ± 0.30 c
Golden	1.64 ± 0.05 c	58.90 ± 4.40 d	24.72 ± 0.77 c	44.61 ± 1.70 d
Annurca	2.13 ± 0.09 b	104.42 ± 5.39 b	29.32 ± 1.08 b	54.40 ± 0.97 b
Significance	***	***	***	***
		***Flesh***		
Limoncella	2.17 ± 0.02 a	44.19 ± 0.52 a	15.31 ± 0.28 a	36.54 ± 1.14 a
Red Delicious	1.21 ± 0,00 c	28.59 ± 0.00 c	8.96 ± 0.06 d	20.75 ± 0.68 c
Golden	1.11 ± 0.01 c	27.96 ± 0.00 c	10.43 ± 0.02 c	17.39 ± 0.56 d
Annurca	1.80 ± 0.02 b	33.20 ± 2.71 b	14.07 ± 0.23 b	25.86 ± 0.36 b
Significance	***	***	***	***
		***Whole fruit***		
Limoncella	2.48 ± 0.05 a	48.60 ± 0.68 a	16.97 ± 0.06 a	38.82 ± 0.43 a
Red Delicious	1.44 ± 0.01 c	38.36 ± 0.78 c	12.29 ± 0.54 c	23.46 ± 0.22 c
Golden	1.26 ± 0.02 c	31.05 ± 0.18 d	11.96 ± 0.07 c	20.44 ± 0.61 d
Annurca	1.94 ± 0.03 b	40.98 ± 1.19 b	15.59 ± 0.11 b	26.63 ± 0.70 b
Significance	***	***	***	***

**Table 3:** Retention time and exact mass spectra data of apple polyphenols investigated by UHPLC-HRMS Orbitrap.

Polyphenols	Molecular Formula	Theoretical Mass [M−H]^-^	Experimental Mass [M−H]^-^	Err [ppm]	Tr (min)
procyanidin b1	C_30_H_26_O_12_	577.13515	577.1358	1.13	7.50
catechin	C_15_H_14_O_6_	289.07176	289.07224	1.66	7.65
chlorogenic acid	C_16_H_18_O_9_	353.0878	353.08798	0.51	8.13
caffeic acid	C_9_H_8_O_4_	179.03498	179.03455	−2.40	8.25
procyanidin b2	C_30_H_26_O_12_	577.13515	577.1355	0.61	8.31
epicatechin	C_15_H_14_O_6_	289.07176	289.07196	0.69	8.51
coumaroyl quinic acid	C_16_H_18_O_8_	337.09289	337.09338	1.45	9.39
rutin	C_27_H_30_O_16_	609.14611	609.14624	0.21	9.78
phloretin xylo-glucoside	C_26_H_32_O_14_	567.17193	567.17206	0.23	9.83
hyperoside	C_21_H_20_O_12_	463.0882	463.085	−6.91	9.89
phloridzin	C_21_H_24_O_10_	435.12967	435.12961	−0.14	10.11
kaempferol-3-O-glucoside	C_21_H_20_O_11_	447.09328	447,09366	0.85	10.28
apigenin-7-glucoside	C_21_H_20_O_10_	431.09837	431.09869	0.74	10.67
phloretin	C_15_H_14_O_5_	273.07684	273.07755	2.60	11.21
epicatechin trimer	C_45_H_38_O_18_	865.19854	865.19928	0.86	8.74
epicatechin tetramer	C_60_H_50_O_24_	1153.26193	1153.26233	0.35	8.84
isorhamnetin glucoside	C_22_H_22_O_12_	477.10385	477.1044	1.15	10.47
isorhamnetin derivative	C_29_H_34_O_15_	621.14611	621.14667	0.90	10.74

**Table 4:** Polyphenols content in flesh and whole fruit of “Limoncella,” “Red Delicious,” “Golden Delicious,” and “Annurca” cultivars detected by HRMS-Orbitrap. Values are expressed in μg g^−1^ (dw). Each value represents the mean of three biological and two technical replicates. Different letters denote a significant difference between cultivars within each part (flesh and peel) by analysis of variance [ANOVA]. Statistical significance was defined as *p* < 0.05, using Tukey’s post hoc test for mean separation. nd: not detected.

	Limoncella	Red Delicious	Golden Delicious	Annurca	Limoncella	Red Delicious	Golden Delicious	Annurca
Polyphenols	*Flesh*				*Whole fruit*			
procyanidin b1	171.12 ± 4.46 a	65.39± 0.75 c	16.46 ± 0.10 d	96.30 ± 1.05 b	174.231 ± 0.65 a	65.099 ± 0.75 b	22.106 ± 1.11 c	94.358 ± 2.22 d
catechin	268.58 ± 5.79 a	137.02 ± 0.73 b	17.16 ± 0.27 d	123.27 ± 0.97 c	256.232 ± 11.33 a	127.500 ± 1.43 b	20.547 ± 0.33 c	116.345 ± 1.33 d
chlorogenic acid	882.76 ± 19.94 a	333.20 ± 10.49 c	580.85 ± 7.30 b	861.79 ± 6.02 a	841.568 ± 2.44 a	314.564 ± 8.22 b	601.332 ± 11.77 c	820.283 ± 14.22 a
caffeic acid	nd	nd	nd	nd	0.43 ± 0.02 a	0.019 ± 0.001 b	0.071 ± 0.001 c	0.400 ± 0.001 a
procyanidin b2	149.98 ± 2.46 b	75.95 ± 6.83 d	104.48 ± 0.0 c	164.74 ± 0.41 a	157.181 ± 1.66 a	84.545 ± 1.33 b	115.327 1.11 c	106.907 ± 0.22 d
epicatechin	279.30 ± 3.66 b	254.70 ± 3.90 c	146.15 ± 6.58 d	361.56 ± 4.68 a	288.600 ± 1.66 a	241.264 ± 0.33 b	170.664 ± 2.44 c	240.306 ± 3.33 b
epicatechin trimer	31.71 ± 3.27 a	32.81 ± 1.03 a	20.89 ± 1.72 b	30.60 ± 0.31 a	34.740 ± 0.45 a	45.821 ± 0.67 b	25.623 ± 0.41 c	32.566 ± 0.77 a
epicatechin tetramer	2.44 ± 0.19 c	4.60 ± 0.05 a	2.90 ± 0.05 b	2.84 ± 0.02 b	4.932 ± 0.04 a	5.566 ± 0.66 b	3.349 ± 0.04 c	1.902 ± 0.03 d
coumaroyl quinic acid	3.45 ± 0–04 c	27.36 ± 0.97 b	26.41 ± 0.67 b	70.27 ± 0.53 a	3.304 ± 0.66 a	24.855 ± 1.11 b	22.61 0.88 2c	65.383 ± 2.77 d
rutin	0.35 ± 0.00 b	0.12 ± 0.00 d	0.26 ± 0.00 b	0.55 ± 0.00 a	2.784 ± 0.03 a	1.311 ± 0.01 b	2.064 ± 0.01a	6.383 ± 0.44 c
phloretin-xylo-glucoside	56.39 ± 0.44 b	19.32 ± 0.13 d	52.83 ± 0.39 c	125.17 ± 0.65 a	58.776 ± 1.44 a	26.623 ± 0.77 b	56.985 ± 1.11 a	108.538 ± 0.88 c
hyperoside	6.32 ± 0.06 b	1.40 ± 0.14 d	11.80 ± 0.30 a	4.91 ± 0.34 c	25.292 ± 0.77 a	45.425 ± 0.71 b	48.186 ± 0.99 b	53.322 ± 1.22 c
phloridzin	44.94 ± 0.07 b	57.68 ± 0.70 a	26.62 ± 0.27 c	10.06 ± 0.27 d	46.754 ± 0.88 a	59.270 ± 1.11 b	27.733 ± 0.66 c	16.989 ± 0.56 d
kaempferolo-3-O-glucoside	9.10 ± 0.18 b	4.31 ± 0.02 d	21.36 ± 0.32 a	5.06 ± 0.01 c	9.552 ± 0.78 a	8.004 ± 0.55 b	24.659 ± 0.66 c	19.197 ± 0.63 d
isorhamnetin-glucoside	0.94 ± 0.01 b	0.93 ± 0.02 b	3.40 ± 0.01 a	0.74 ± 0.05 c	1.598 ± 0.03 a	3.154 ± 0.02 b	3.060 0.05 b	3.599 ± 0.01 c
apigenin-7-glucoside	0.15 ± 0.00 b	0.02 ± 0.00 c	0.34 ± 0.01 a	0.14 ± 0.00 b	0.222 ± 0.01a	0.087 ± 0.01 b	0.301 ± 0.05 c	0.275 ± 0.01 d
phloretin	0.30 ± 0.01 c	0.96 ± 0.01 a	0.32 ± 0.00 c	0.57 ± 0.00 b	0.315 ± 0.002 a	0.740 ± 0.001 b	0.358 ± 0.001 a	0.779 ± 0.02b
isorhamnetin derivative	nd	nd	nd	nd	0.001 ± 0.0001a	0.001 ± 0.0001a	nd	0.440 ± 0.001b
Total polyphenols	1907.84 ± 39.55 a	1015.76 ± 24.04 b	1032.23 ± 14.06 b	1858.55 ± 13.46 a	1906.520 ± 21.22 a	1053.848 ± 12.22 b	1144.976 ± 9.11 b	1687.973 ± 24.11 c

**Table 5:** Polyphenols content in the peels of “Limoncella,” “Red Delicious,” “Golden Delicious,” and “Annurca” cultivars detected by HRMS-Orbitrap. Values are expressed in μg/g (dw). Each value represents the mean of three biological and two technical replicates. Different letters denote a significant difference between cultivars by analysis of variance [ANOVA]. Statistical significance was defined as *p* < 0.05, using Tukey’s post hoc test for mean separation. nd: not detected.

	Limoncella	Red Delicious	Golden Delicious	Annurca
Polyphenols	*Peel*			
procyanidin b1	133.02 ± 1.50 a	44.38 ± 0.52 c	19.52 ± 0.55 d	91.13 ± 2.78 b
catechin	118.59 ± 0.33 a	52.44 ± 0.36 c	40.32 ± 0.62 d	84.35 ± 2.10 b
chlorogenic acid	340.37 ± 15.52 b	127.38 ± 10.05 c	313.13 ± 1.97 b	476.68 ± 6.41 a
caffeic acid	0.12 ± 0.00 a	nd	nd	0.04 ± 0.01 b
procyanidin b2	174.87 ± 18.35 c	195.25 ± 11.58 b	156.64 ± 3.29 c	262.31 ± 2.71 a
epicatechin	367.47 ± 8.46 a	281.59 ± 1.39 c	327.61 ± 7.74 b	342.64 ± 17.87 ab
epicatechin trimer	58.15 ± 7.20 b	91.64 ± 2.11 a	57.17 ± 1.87 b	60.54 ± 1.56 b
epicatechin tetramer	8.19 ± 0.35 b	15.87 ± 0.96 a	7.11 ± 0.13 b	8.22 ± 0.32 b
coumaroyl quinic acid	2.44 ± 0.07 b	2.58 ± 0.07 b	7.80 ± 0.97 b	28.21 ± 3.55 a
rutin	31.80 ± 0.47 b	15.21 ± 0.25 c	13.83 ± 0.98 c	66.06 ± 9.03 a
phloretin-xylo-glucoside	105.72 ± 1.34 b	76.37 ± 1.68 b	88.23 ± 1.81 b	210.85 ± 21.07 a
hyperoside	213.77 ± 2.75 c	427.82 ± 3.20 ab	401.35 ± 7.52 b	468.18 ± 32.74 a
phloridzin	72.47 ± 1.77 c	101.51 ± 3.30 a	66.39 ± 1.82 c	92.06 ± 2.53 b
kaempferolo-3-O-glucoside	73.38 ± 0.21 c	90.97 ± 0.81 b	124.53 ± 7.19 a	87.31 ± 6.84 c
isorhamnetin-glucoside	5.21 ± 0.11 c	28.10 ± 0.08 b	5.07 ± 0.29 c	72.59 ± 6.95 a
apigenin-7-glucoside	1.48 ± 0.01 a	0.80 ± 0.00 c	1.59 ± 0.06 a	1.12 ± 0.04 b
phloretin	0.51 ± 0.01 c	1.55 ± 0.07 b	0.62 ± 0.03 c	3.38 ± 0.07 a
isorhamnetin derivative	0.28 ± 0.00 b	0.24 ± 0.00 b	nd	5.26 ± 0.23 a
Total polyphenols	1707.84 ± 12.53 b	1553.71 ± 25.48 d	1630.91 ± 19.76 c	2360.93 ± 8.89 a

**Table 6:** Concentrations of polyphenols released upon in vitro digestion of apple sample (flesh, peel, and whole fruit). Polyphenols were detected by HRMS-Orbitrap and values are expressed in μg/g (dw). Each value represents the mean ± SD of three replicates. For each line, different letters indicate significantly different values (*p* < 0.05) according to Tukey’s test. NF: not found.

Compound	Annurca (Whole Fruit)			Annurca Flesh			Annurca Peel		
	SDP	SCP	Total	SDP	SCP	Total	SDP	SCP	Total
Flavanols	0.041 ± 0.001 a	2.770 ± 0.01 b	2.811 ± 0.02 b	0.001 ± 0.0001 c	4.723 ± 0.01 d	4.723 ± 0.21 d	NF	14.341 ± 0.33 e	14.341 ± 0.37 e
Procyanidins	NF	1.544 ± 0.02 a	1.544 ± 0.02 a	NF	1.454 ± 0.03 a	1.454 ± 0.03 a	NF	7.023 ± 0.22 b	7.023 ± 0.22 b
Hydroxycinnamic acids	119.557 ± 0.88 a	92.101 ± 1.33 b	211.658 ± 2.33 c	39.607 ± 1.45 d	72.408 ± 3.33 e	112.014 ± 1.44 f	13.479 ± 1.41 g	52.187 ± 0.44 h	65.666 ± 1.33 i
Flavonols	44.712 ± 0.78 a	17.270 ± 0.91 b	61.982 ± 1.88 c	1.029 ± 0.02 d	0.931 ± 0.02 d	1.960 ± 0.08 e	52.159 ± 1.33 f	78.294 ± 2.33 g	130.453 ± 4.55 h
Dihydrochalcones	31.880 ± 0.66 a	12.880 ± 1.66 b	44.760 ± 0.83 c	12.226 ± 1.63 b	9.739 ± 0.56 d	21.965 ± 0.67 e	19.223 ± 0.87 e	28.678 ± 0.76 f	47.900 ± 1.76 g
Total PPs	196.189 ± 11.12 a	126.565 ± 7.34 b	322.754 ± 15.76 c	52.862 ± 3.44 d	89.254 ± 2.67 e	142.116 ± 1.67 f	84.860 ± 0.56 g	180.522 ± 11.78 h	265.382 ± 7.65
Compound	Golden Delicious (whole fruit)			Golden Delicious (flesh)			Golden Delicious (peel)		
	SDP	SCP	Total	SDP	SCP	Total	SDP	SCP	Total
Flavanols	0.143 ± 0.02 a	1.512 ± 0.04 b	1.655 ±0.03 c	NF	0.108 ±0.04 d	0.108 ± 0.04 d	NF	1.712 ±0.03 e	1.712 ± 0.03 e
Procyanidins	NF	0.796 ±0.01 a	0.796 ± 0.01 a	NF	0.035 ±0.002 b	0.035 ±0.002 b	NF	3.679 ±0.03 c	3.679 ±0.03 c
Hydroxycinnamic acids	83.120 ± 1.56 a	75.132 ± 2.44 b	158.252 ± 3.67 c	3.860 ± 0.77 d	5.587 ± 0.99 e	9.447 ± 0.88 f	1.401 ± 0.05 g	6.314 ± 0.14 h	7.715 ± 0.87 i
Flavonols	22.641 ± 0.99 a	5.695 ± 1.44 b	28.336 ± 0.88 c	1.913 ± 0.01 d	2.947 ± 0.01 e	4.859 ± 0.44 f	93.074 ± 1.66 g	67.314 ±2.77 h	160.388 ± 0.63 i
Dihydrochalcones	11.723 ± 0.88 a	4.641 ± 0.77 b	16.364 ± 0.71 c	0.211 ± 0.03 d	0.517 ± 0.01 e	0.728 ± 0.05 f	5.255 ± 0.03 g	3.226 ± 0.11 h	8.481 ± 0.92 i
Total PPs	117.627 ± 1.55 a	87.776 ± 2.55 b	205.403 ± 5.56 c	5.983 ± 0.54 d	9.194 ± 0.65 e	15.178 ± 0.43 f	99.730 ± 1.66 g	82.245 ±1.87 h	181.976 ± 3.44 i
Compound	Limoncella (whole fruit)			Limoncella (flesh)			Limoncella (peel)		
	SDP	SCP	Total	SDP	SCP	Total	SDP	SCP	Total
Flavanols	0.621 ± 0.01 a	9.843 ±0.11 b	10.464 ± 0.55 c	0.003 ± 0.001 d	5.557 ± 0.61 e	5.560 ± 0.65 e	NF	16.449 ± 1.33 f	16.449 ± 1.33 f
Procyanidins	NF	5.997 ± 0.34 a	5.997 ± 0.34 a	0.001 ± 0.0001 b	1.549 ± 0.02 c	1.550 ± 0.02 c	NF	8.359 ± 0.76 d	8.359 ± 0.76 d
Hydroxycinnamic acids	97.438 ± 2.55 a	113.302 ± 9.11 b	210.740 ± 3.76 c	45.980 ± 2.66 d	120.023 ± 2.65 e	166.002 ± 3.11 f	14.352 ± 0.56 g	61.785 ± 2.76 h	137.922 ± 2.79 i
Flavonols	58.800 ± 1.67 a	19.554 ± 2.76 b	78.354 ± 3.65 c	1.016 ± 0.02 d	0.337 ± 0.03 e	1.353 ± 0.06 f	56.650 ± 2.76 g	76.914 ± 2.69 h	133.564 ± 3.71 i
Dihydrochalcones	37.678 ± 1.87 a	17.706 ± 0.91 b	55.384 ± 2.54 c	19.201 ± 0.65 d	10.578 ± 0.54 e	29.779 ± 1.62 f	3.759 ± 0.23 g	28.371 ± 1.81 h	32.130 ± 0.65 i
Total PPs	194.538 ± 2.87 a	166.402 ± 2.89 b	360.940 ± 3.91 c	66.201 ± 0.81 d	138.044 ± 2.97 e	204.245 ± 4.11 f	74.761 ± 3.44 g	191.878 ± 1.33 h	266.639 ± 5.79 i
Compound	Red Delicious (whole fruit)			Red Delicious (flesh)			Red Delicious (peel)		
	SDP	SCP	Total	SDP	SCP	Total	SDP	SCP	Total
Flavanols	0.448 ± 0.03 a	1.091 ± 0.04 b	1.538 ± 0.01 c	NF	0.261 ± 0.04 d	0.261 ± 0.04 d	NF	2.106 ± 0.05 e	2.106 ± 0.05 e
Procyanidins	NF	0.608 ± 0.03 a	0.608 ± 0.03 a	NF	0.279 ± 0.01 b	0.279 ± 0.01 b	NF	3.943 ± 0.14 c	3.943 ± 0.14 c
Hydroxycinnamic acids	85.997 ± 3.34 a	46.019 ± 1.67 b	132.016 ± 2.66 c	2.055 ± 0.33 d	2.757 ± 0.11 e	4.812 ± 0.77 f	2.571 ± 0.66 g	5.059 ± 0.88 h	7.630 ± 1.63 i
Flavonols	8.426 ± 0.56 a	2.095 ± 0.77 b	10.520 ± 0.91 c	0.164 ± 0.01 d	0.644 ± 0.02 e	0.807 ± 0.02 f	73.498 ± 2.67 g	46.954 ± 1.89 h	120.453 ± 3.91 i
Dihydrochalcones	9.056 ± 1.22 a	2.971 ± 0.91 b	12.027 ± 0.56 c	0.059 ±0.004 d	0.253 ± 0.02 e	0.313 ± 0.01 f	17.002 ± 0.91 g	9.563 ± 1.56 h	26.566 ± 0.87 i
Total PPs	103.926 ± 2.75 a	52.783 ± 3.67 b	156.709 ± 4.56 c	2.278 ± 0.95 d	4.194 ± 0.56 e	6.472 ± 0.95 f	93.071 ± 2.96 g	67.626 ± 2.77 h	160.697 ± 3.83 i

**Table 7:** % of polyphenols released in the soluble duodenal phase (SDP) and in soluble colonic phase (SCP) upon enzymatic digestion of apple sample. Each value represents the mean of three replicates. Different letters denote a significant difference (*p* < 0.05 according to Tukey’s test) between cultivars within flesh, peel, and whole fruit.

% of Polyphenols Released in the SDP			% of Polyphenols Released in the SCP		
Annurca			Annurca		
Whole	Flesh	Peel	Whole	Flesh	Peel
60.786 ± 0.121 a	37.197 ± 0.812 a	31.977 ± 0.123 a	39.214 ± 0.821 a	62.803 ± 1.012 a	68.023 ± 0.231 a
Golden Delicious			Golden Delicious		
Whole	Flesh	Peel	Whole	Flesh	Peel
57.267 ± 0.123 b	39.423 ± 0.161 b	54.804 ± 0.341 b	42.733 ± 0.211 b	60.57 ± 0.236 b	45.19 ± 0.439 b
Limoncella			Limoncella		
Whole	Flesh	Peel	Whole	Flesh	Peel
53.898 ± 0.714 c	32.412 ± 0.581 c	28.038 ± 0.619 c	46.102 ± 1.371 c	67.588 ± 0.912 c	71.96 ± 0.459 c
Red Delicious			Red Delicious		
Whole	Flesh	Peel	Whole	Flesh	Peel
66.318 ± 1.812 d	35.200 ± 0.172 d	57.917 ±0.291 d	33.68 ± 1.671 d	64.8 ± 0.291 d	42.08 ±0.457 d

**Table 8:** Antioxidant activity measured with DPPH assay in Limoncella, Red Delicious, Golden Delicious, and Annurca upon in vitro digestion of apple samples (flesh, peel, and whole fruit). Each value represents the mean ± SD of three replicates. For each line, different letters indicate significantly different values (*p* < 0.05) according to Tukey’s test.

Apple Samples	SDP	SCP	TOTAL
	TEAC (mmol trolox/kg DW)		
**Annurca**			
Whole fruit	4.480 ± 0.71 a	7.854 ±0.23 b	12.334 ± 0.67 c
Flesh	3.920 ± 0.12 a	7.943 ± 0.23 b	11.863 ± 0.24 c
Peel	9.125 ± 0.67 a	15.580 ± 0.21 b	24.705 ± 1.76 c
**Golden Delicious**			
Whole fruit	3.610 ± 0.72 a	5.861 ± 1.33 b	9.471 ± 0.56 c
Flesh	3.152 ± 0.65 a	5.438 ± 0.43 b	8.590 ± 0.12 c
Peel	7.876 ± 0.11 a	10.785 ± 0.32 b	18.661 ± 0.72 c
**Limoncella**			
Whole fruit	2.720 ± 0.81 a	12.653 ± 1.76 b	15.373 ± 1.11 c
Flesh	4.127 ± 0.67 a	9.654 ± 1.56 b	13.781 ± 0.88 c
Peel	9.873 ± 1.77 a	18.743 ± 0.99 b	28.616 ± 1.61 c
**Red Delicious**			
Whole fruit	4.012 ± 0.71 a	4.874 ± 0.12 b	8.886 ± 0.91 c
Flesh	3.580 ± 0.61 a	4.870 ± 0.93 b	8.450 ± 0.45 c
Peel	8.010 ± 0.65 a	13.650 ± 0.56 b	21.660 ± 1.41 c

## Data Availability

The data presented in this study are available on request from the corresponding author.

## Supplementary Materials

The following are available online at https://www.mdpi.com/article/10.3390/antiox10040541/s1, Table S1: Concentrations of individual polyphenols released upon in vitro digestion of apple sample, flesh (F), peel (P) and whole fruit (W). Polyphenols were detected by HRMS-Orbitrap and values are expressed in μg/g (dw).
